# Ideal Lymph Node Number for Ovarian Malignancies

**DOI:** 10.7759/cureus.20869

**Published:** 2022-01-02

**Authors:** İbrahim Karadağ, Serdar Karakaya

**Affiliations:** 1 Medical Oncology, Health Sciences University, Ankara Dr. Abdurrahman Yurtaslan Oncology Training and Research Hospital, Ankara, TUR; 2 Medical Oncology, Health Sciences University, Atatürk Chest Diseases and Chest Surgery Training and Research Hospital, Ankara, TUR

**Keywords:** overall survival (os), prognosis, ovarian cancer, lymph node number, lymph node disection

## Abstract

Objective: Although there are studies in which the ideal number of lymph nodes for early-stage ovarian cancer is specified, no study has been found on the number of lymph nodes that should ideally be removed by systematic lymph node dissection, including advanced-stage patients. The present study was aimed to retrospectively investigate the number of lymph nodes that need to be removed to detect lymph node positivity and the effect of this number on prognosis.

Methodology: A total of 155 patients over the age of 18 who were diagnosed with ovarian cancer without secondary malignancy and who underwent surgical lymph node dissection were included in the study between 2015 and 2020.

Results: A total of 155 patients underwent lymphadenectomy and the median number of removed lymph nodes was 24. Lymph node positivity was detected in 72 (46.4%) of these patients, while the median number of positive lymph nodes was 4 in the lymph node-positive group. A statistically significant positive correlation was found between the number of lymph nodes removed and the median overall survival (OS) (r = 0.546, p<0.001). At the same time, when the number of 24 lymph nodes, which is the median number of lymph nodes removed and the value found to detect lymph node positivity in the receiver operating characteristic (ROC) curve, is taken as cut off; mean OS was found to be statistically significantly higher in the group with adequate lymph node dissection compared to the group with insufficient lymph node dissection (46.46±35.22 vs 22.33±21.43; p < 0.001, respectively).

Conclusion: it was shown that more than 24 lymph nodes are required for adequate lymph node dissection in the patients included in the study, and thus it can contribute positively to the prognosis. With the support of more comprehensive and prospective studies conducted on this subject to this study, clearer data will emerge about the number of lymph nodes that should be removed in an ideal surgery.

## Introduction

Ovarian cancer is the most common cause of death from gynecological cancers and ranks fifth in cancer deaths in women [1]. Ovarian cancer has different tumor biology and metastasis pattern which is mainly peritoneal, lymphovascular spread, and rarely hematological spread. Essentially, the role of complete tumor resection of intraperitoneal disease is well defined, and the main surgical target is optimal debulking, defined as no or <1 cm macroscopic residue in ovarian cancer [2]. Age, tumor stage, and residual disease are the main prognostic factors in ovarian cancer [3,4]. Pelvic and paraaortic lymph node dissection is important for surgical staging of ovarian cancer as well as cervical and endometrial cancer. However, the effect of lymph node metastasis on residual tumor status has not been fully clarified, and the prognostic value of lymph node metastases is less well defined. The pelvic lymph nodes are anatomically below the common iliacs and are the external, internal, obturator, sacral and paraaortic lymph nodes. The paraaortic lymph nodes are the region from the inferior mesenteric artery to the middle of the common iliac. Studies addressing the therapeutic value of extensive pelvic and paraaortic lymphadenectomy in patients with ovarian cancer are limited. In retrospective analyzes, lymphadenectomy has been shown to make a positive contribution, especially in patients with optimal debulking ovarian cancer and early-stage ovarian cancer [5,6]. However, in a prospective study comparing those with systematic lymph node dissection and those with extended lymph node dissection, including patients with macroscopic residues, no contribution to overall survival (OS) was demonstrated [7]. 

It is known that lymph node positivity in ovarian cancer staging increases the stage and worsens the expected survival [8]. Similarly, detection of lymph node positivity in colorectal and gastric cancers increases the stage and worsens the prognosis. The Eighth Edition AJCC Cancer Staging Manual has determined the ideal number of lymph node dissections to be performed with systematic dissection for an ideal staging of these tumors (12 lymph nodes for colorectal, 15 lymph nodes for stomach) [8]. Although there are studies in which the ideal number of lymph nodes for early-stage ovarian cancer is specified, no textbook information or study has been found on the number of lymph nodes that should ideally be removed by systematic lymph node dissection, including advanced-stage patients [9]. In this study, we aim to retrospectively investigate the number of lymph nodes that need to be removed to detect lymph node positivity and the effect of this number on prognosis.

## Materials and methods

A total of 171 patients with ovarian cancer who were followed up in our oncology center between 2015 and 2020 were retrospectively screened. A total of 155 patients over the age of 18 who were diagnosed with ovarian cancer without secondary malignancy and who underwent surgical lymph node dissection were included in the study. The files of the patients were investigated retrospectively and their demographic data, time of progression, date of death or last control were recorded, and survival and disease-free survival (DFS) times were calculated. DFS was defined as the time from surgery to the date of progression or the last control, while OS was defined as the time from diagnosis to death or the date of the last control. Approval for the study was obtained from the local ethics committee of Health Sciences University, Ankara Dr. Abdurrahman Yurtaslan Oncology Training and Research Hospital (2021-08/1348).

Statistical analysis

Statistical analyzes were performed with SPSS software, version 25.0 (SPSS, Chicago, IL, USA). Mann Whitney U test was used to compare nonparametric data and student T test was used to compare parametric data. Chi-square or Fisher's exact test was used to compare categorical data. Kaplan-Meier method was used for survival analysis and log-rank test was performed for comparisons between groups. The optimum cut-off point for lymph node dissection was determined using a receiver operating characteristic (ROC) curve. Prognostic factors affecting OS were determined by multivariate analysis with the Cox proportional hazards model. P value <0.05 was considered as statistically significant.

## Results

The median age of 171 patients in the study was 56 (48-65) at the time of diagnosis, and 73.1% were postmenopausal. At the time of diagnosis, 71.9% (n=123) of the patients were in stage 3 and 4, while 92.3% (n=155) underwent surgery. Optimal surgical resection was performed in 85.8% (n=133) of the patients who underwent surgery. Preoperative neoadjuvant three cycles of platinum-taxane combination therapy were given to 22.8% (n=39) of the patients. Hyperthermic intraperitoneal chemotherapy (HIPEC) with cisplatin was administered to 11.7% (n=20) of the patients in the study. High grade serous (n=130) cancers were the most common pathological subtype with 76%; 81.3% (n=139) of the patients had received adjuvant median five cycles of platinum-taxane-based chemotherapy. The basic characteristics of the patients are summarized in Table 1.

**Table 1:** Baseline characteristics of patients

Variables	N	Percent %
Age (years)	56 (IQR 48-65)	
Menopausal Status		
Premenopausal	34	19.9%
Postmenopausal	125	73.1%
Unknown	12	7%
Surgery Type		
Optimal surgery	133	77.8%
Suboptimal surgery	22	12.8%
Inoperable	16	9.4%
TNM (Tumor, Node, Metastasis) Stage		
Stage 1	27	15.8%
Stage 2	18	10.5%
Stage 3	103	60.2%
Stage 4	20	11.7%
Unknown	3	1.8%
Histologic Type		
Serous	130	76%
Non-serous	41	24%
Treatment Type		
Neoadjuvant Chemotheraphy	39	22.8%
Adjuvant Chemotheraphy	139	81.3%
Hyperthermic Intraperitoneal chemotherapy	20	11.7%

**Figure 1:**
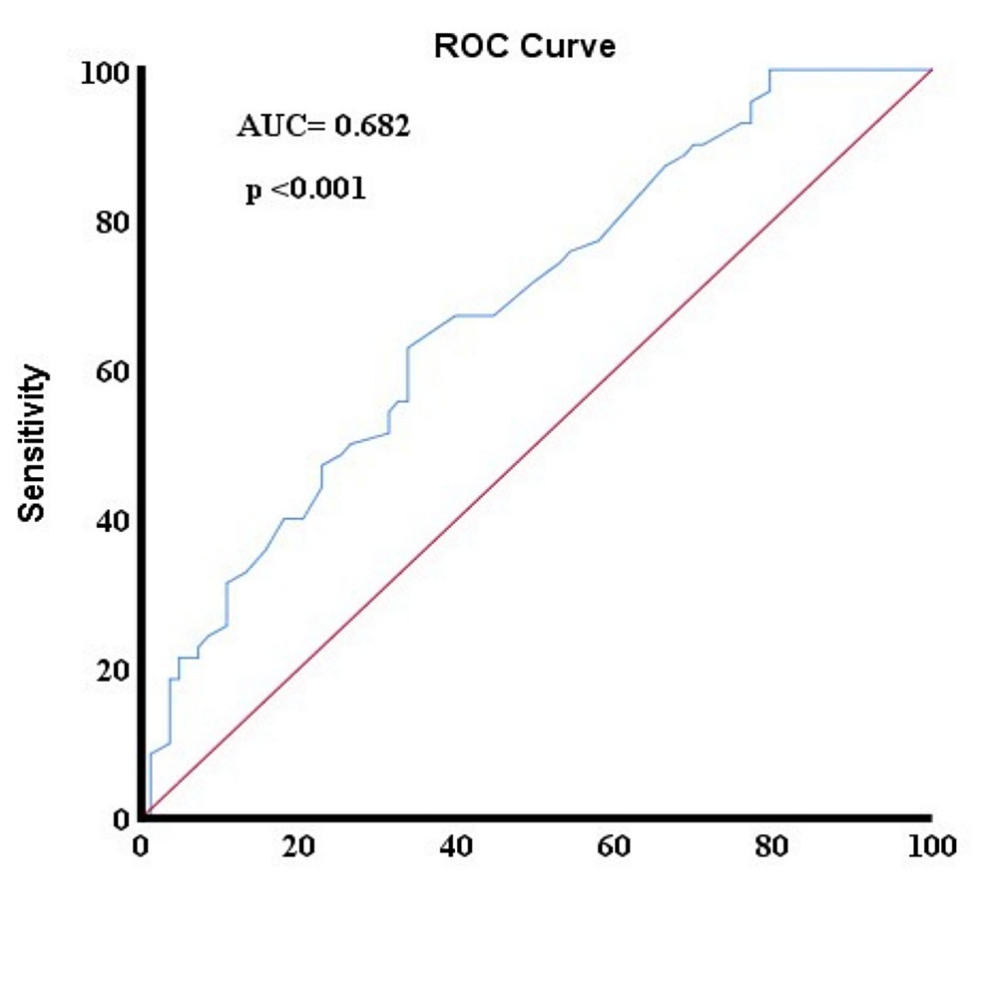
ROC curve for positive lymph node ROC: receiver operating characteristic; AUC: area under curve.

In the present study, 155 patients underwent lymphadenectomy and the median number of removed lymph nodes was 24 (interquartile range (IQR) 16-37). Lymph node positivity was detected in 72 (46.4%) of these patients, while the median number of positive lymph nodes was 4 (IQR 2-8) in the lymph node-positive group. Optimal cut-off points for the number of removed lymph nodes were analyzed using a ROC curve. As a result, the optimal lymph node cut-off number required to detect lymph node metastasis was determined as 24 with 62.9% sensitivity and 66.3% specificity (area under curve = 0.682; p < 0.001) (Figure 1). Patients with ≤24 lymph nodes removed (insufficient lymph node dissection) were defined as Group 1 and those with <24 lymph nodes removed (adequate lymph node dissection) were defined as Group 2. There was no statistically significant difference between the two groups in terms of age (p= 0.447) but stage 4 disease was more common in Group 2 (p<0.001). Again, there was no significant difference between the groups in terms of the distribution of histopathological subgroups (p=0.276).

In the present study, the median follow-up duration was 27 months (IQR 15-43). At the time of analysis, 62.6% of patients were alive. In the study, the median OS was 53 months (40.61-65.38, 95% CI), while the five-year OS was 43.6%. A statistically significant positive correlation was found between the number of lymph nodes removed and the median OS (r = 0.546, p<0.001). At the same time, when the number of 24 lymph nodes, which is the median number of lymph nodes removed and the value found to detect lymph node positivity in the ROC curve, is taken as cut off; mean OS was found to be statistically significantly higher in the group with adequate lymph node dissection compared to the group with insufficient lymph node dissection (46.46±35.22 vs 22.33±21.43; p < 0.001, respectively). Median DFS was calculated as 20 months (IQR 12-33) in the study. A statistically significant positive correlation was found between the number of lymph nodes removed and DFS (r=0.196, p =0.015). Mean DFS was found to be numerically higher in the group with adequate lymph node dissection compared to the group with insufficient lymph node dissection (30.98±29.76 vs 24.82±31.39; p = 0.217, respectively).

## Discussion

In the present study, it was shown that removal of more than 24 lymph nodes in 155 patients who were diagnosed with ovarian cancer and operated on contributed to OS.

While the patients in the present study were slightly younger than the literature, the majority of the patients were postmenopausal and at stage 3 disease, similar to the literature [10]. Lymph node dissection is one of the basic parts of staging in ovarian cancer and its purpose is to try to detect the presence of positive lymph nodes. As a result, it contributes to decision making about disease prognosis and treatment. It is known that with lymph node positivity, the stage rises and the prognosis worsens. This has been supported by studies showing that as the rate of positive lymph nodes increases, survival decreases [11,12]. However, in these studies, no information was given on the number of lymph nodes that should be removed to detect lymph node positivity and the number of lymph nodes that should ideally be removed. The ideal number of lymph nodes to be removed has been specified in the guidelines for colorectal cancers and gastric cancer, and similarly, studies have been conducted on the number of lymph nodes that should be removed in endometrial cancer [8,13]. Considering the information on these tumors, in the present study, unlike the ovarian cancer studies mentioned above, the number of lymph nodes required to be removed to detect a positive lymph node was found to be >24. Removal of more than 24 lymph nodes has been shown to contribute positively to OS. Although this positive contribution did not reach statistical significance in DFS, it was found to be high numerically. The significant positive correlation between the number of lymph nodes removed in the study and OS and DFS suggests that in studies to be conducted by increasing the number of patients, the contribution of lymph node removal to DFS may reach statistical significance. It is known that survival decreases with increasing stage in ovarian cancer [8,14]. In the present study, the mean OS was found to be higher in the group with adequate lymph node dissection, although stage 4 disease was statistically significantly higher than in the group with insufficient lymph node dissection. This suggests that staging could not be fully completed in the group with insufficient lymph node dissection. Based on this, removing ≤ 24 lymph nodes in ovarian cancer can be considered a bad risk factor. On the other hand, as the number of removed lymph nodes increases, the morbidity that this will bring should also be taken into consideration.

There are some limitations to the study. Perioperative morbidity and complications of the lymphadenectomy could not be reported. Due to the fact that it is a retrospective study, the risk of bias may be caused by missing data. It is a single-center study with a relatively small number of patients.

## Conclusions

It was shown that more than 24 lymph nodes are required for adequate lymph node dissection in the patients included in the study, and thus it can contribute positively to the prognosis and may help clarify the surgical staging. With the support of more comprehensive and prospective studies to be conducted on this subject, clearer data will emerge about the number of lymph nodes that should be removed in an ideal surgery.
